# Impact of inoculum effect on *in vitro* activity of intermittent and continuous piperacillin-tazobactam infusion against ESBL-producing *Escherichia coli* in a hollow-fiber infection model

**DOI:** 10.1128/spectrum.01457-26

**Published:** 2026-10-13

**Authors:** Marion Giry, Charles Baulier, Fabien Lamoureux, Manuel Etienne, Nicolas Nessi, François Gravey, Kévin Alexandre

**Affiliations:** 1 Department of Medical Intensive Care, Univ Rouen Normandie, Univ de Caen Normandie, INSERM, DYNAMICURE UMR 1311, CHU de Rouen 27003, Rouen, France; 2 Department of Anaesthesiology, Critical Care and Perioperative Medicine, Univ Rouen Normandie, Univ de Caen Normandie, INSERM, DYNAMICURE UMR 1311, CHU de Rouen 27003, Rouen, France; 3 Department of Pharmacology, CHU de Rouen, Rouen, France; 4 Toxlab Laboratory, Paris, France; 5 Department of Infectious Diseases, Univ Rouen Normandie, Univ de Caen Normandie, INSERM, DYNAMICURE UMR 1311, CHU de Rouen 27003, Rouen, France; 6 Normandie Univ, UNICAEN, UNIROUEN, INSERM, DYNAMICURE UMR 1311, Caen, France; 7 Department of Microbiology, Univ de Caen Normandie, Univ Rouen Normandie, INSERM, DYNAMICURE UMR 1311, CHU de Caen 27003, Caen, France; Maryland Department of Health, Baltimore, Maryland, USA

**Keywords:** *Escherichia coli*, gram-negative bacterial infections, β-lactam resistance, pharmacokinetics, hollow-fiber infection model, ESBL

## Abstract

Use of piperacillin-tazobactam (TZP) in the treatment of severe extended-spectrum β-lactamase (ESBL)-producing *Escherichia coli* (ESBL-EC) infections is controversial. Two features may explain treatment failures: the inoculum effect and suboptimal pharmacokinetic/pharmacodynamic (PK/PD) target attainment, particularly in critically ill patients. To study the combined impact of the inoculum effect and a PK/PD optimization on the *in vitro* efficacy of TZP against ESBL-EC infections. Five-day hollow-fiber infection model simulating TZP pharmacokinetics in critically ill patients was performed: two TZP infusion regimens (4/0.5 g q6h over 30 min, and 16/2 g q24h continuous infusion), three ESBL-EC clinical strains: *1146* (*bla*_CTX-M-15_, MIC = 2 mg/L), *3383* (*bla*_CTX-M-15_ + *bla*_TEM-1_, MIC = 4 mg/L), and *2262* (*bla*_CTX-M-15_ + *bla*_OXA-1_, MIC = 8 mg/L), and two initial inocula (5 and 7 log_10_ CFU/mL). Intermittent infusion was associated with systematic bacterial regrowth within 24 h, consistent with failure to reach the 100%fT >4× MIC threshold. Continuous infusion resulted in undetectable bacterial count for the strains *3383* (MIC = 4 mg/L, low inoculum) and *1146* (MIC = 2 mg/L, low and high inocula). For strain *3383*, the inoculum effect was demonstrated by total bacterial killing in continuous infusion with low inoculum, whereas early regrowth (>10 log_10_ CFU/mL) with resistance emergence (MIC > 256 mg/L) was observed with high inoculum. Strain *2262* (MIC = 8 mg/L) exhibited systematic treatment failure regardless of infusion mode or inoculum size. Continuous infusion of TZP may be considered as a carbapenem-sparing agent in ESBL-EC infection with a TZP MIC ≤ 2 mg/L regardless of inoculum, or a TZP MIC of 4 mg/L at low inoculum. TZP should not be used for strains with an MIC of 8 mg/L or suspected OXA-1 co-production, irrespective of the infusion mode.

**IMPORTANCE:**

Infections caused by extended-spectrum β-lactamase (ESBL)-producing *Escherichia coli* represent a growing therapeutic challenge, particularly in critically ill patients. Carbapenems remain the reference treatment, but their overuse drives resistance emergence. Piperacillin-tazobactam is a potential carbapenem-sparing agent, but its clinical efficacy remains debated, partly due to the inoculum effect (a reduction in antibiotic activity at high bacterial densities) and suboptimal dosing regimens. Using a hollow-fiber infection model simulating the pharmacokinetics of critically ill patients, this study demonstrates that continuous infusion of piperacillin-tazobactam can overcome the inoculum effect under specific conditions, defined by the MIC of the infecting strain and the estimated bacterial density. These findings support a stratified approach to piperacillin-tazobactam use in severe ESBL-producing *E. coli* infections, based on MIC, inoculum size, and β-lactamase profile, and may help clinicians identify patients who could safely receive piperacillin-tazobactam instead of a carbapenem.

## INTRODUCTION

Infections caused by extended-spectrum β-lactamase (ESBL)-producing *Enterobacterales* (ESBL-E) are a global threat to human health. Annually, 50,000–100,000 deaths worldwide are attributed to third-generation cephalosporin (3GC)*-*resistant *Escherichia coli* (1). Compared with infection due to 3GC-susceptible strains, ESBL-E infections are associated with increased mortality, especially in critically ill patients (2). International guidelines recommend using carbapenems for ESBL-E infections (3, 4). However, due to the emergence of carbapenem resistance, alternatives have been studied, including piperacillin-tazobactam (TZP) (5).

Numerous retrospective studies suggest that TZP could be an alternative to carbapenems for ESBL-E infections (6–8). However, the only prospective randomized study to date (MERINO trial) reported that definitive treatment with TZP compared to meropenem did not result in noninferior 30-day mortality (9). Moreover, TZP MIC > 16 mg/L, measured by broth microdilution, was found to best predict mortality risk (10). Factors that, among others, could explain the MERINO study’s results were unfavorable pharmacokinetic (PK) profiles in patients treated with TZP, β-lactamase profile, and inoculum effect. TZP was administered at 4.5 g/6 h in 30 min, while prolonged or continuous infusion is recommended to reach pharmacokinetic/pharmacodynamic (PK/PD) targets, particularly in critically ill patients (11). Previous studies have demonstrated that prolonged or continuous TZP infusion is associated with higher clinical cure rates and lower mortality, compared with intermittent infusion (12). A MERINO *post hoc* analysis showed that many isolates carrying both OXA-1 and ESBL, classified as susceptible by routine susceptibility testing, were non-susceptible by the reference broth microdilution method, likely because their TZP MIC commonly ranged from 8 to 32 µg/mL. Excluding these isolates attenuated the difference in 30-day mortality between TZP and meropenem (10). The inoculum effect is defined by a four- or eightfold MIC increase when the antibiotic is exposed to a high density of bacteria (e.g., ≥10^7^ vs 10^5^ CFU/mL) (13). The activity of carbapenems is less altered by the inoculum effect, although exceptions have been reported, particularly in carbapenemase-producing *Enterobacterales* (14), while the TZP activity is significantly affected *in vitro* and *in vivo* (15, 16). Therefore, TZP treatment efficacy against ESBL-E could be compromised in serious infections associated with high bacterial density (e.g., pneumonia or intra-abdominal infections) compared to low bacterial density (e.g., urinary tract infections). However, it is unknown whether TZP PK optimization with continuous infusion could overcome the inoculum effect in serious infections due to ESBL-E with various TZP MIC. The hollow-fiber infection model (HFIM), a dynamic *in vitro* two-compartment PK/PD model, was selected because it allows unraveling some of the MERINO confounders (PK optimization, known complete β-lactamase profile, and inoculum effect) under controlled conditions (17).

The aim of this study was to evaluate TZP *in vitro* activity against ESBL-producing *Escherichia coli* (ESBL-EC) infections in a HFIM according to infusion regimen, inoculum, and strain MIC range.

## MATERIALS AND METHODS

### Antibiotics and media

Cation-adjusted Mueller-Hinton (CAMH) broth and agar (BD, Sparks, MD, USA) were used for both HFIM experiments and preparation of drug-free and antibiotic-enriched plates.

Piperacillin sodium salt (Sigma-Aldrich, reference P8396) and tazobactam (Sigma-Aldrich, reference PHR1686) powder were used for *in vitro* susceptibility testing and the preparation of antibiotic-enriched agar plates (piperacillin at fourfold the initial MIC of the studied bacterial strain, fixed tazobactam concentration of 4 mg/L). A clinical-use formulation of TZP (piperacillin-tazobactam 4 g/0.5 g, Panpharma) was used for the HFIM experiments.

### Bacterial strains

Three clinical ESBL-EC strains, obtained from patients with urinary tract infections, were studied: *E. coli 1146*, *E. coli 3383*, and *E. coli 2262*. These strains were selected based on their various TZP MIC.

### *In vitro* susceptibility studies

TZP MICs were measured in triplicate at a standard inoculum (10^5^ CFU/mL) using CLSI guidelines for broth microdilution method (18). Quality control was performed routinely using *E. coli* ATCC 35218 strain. Susceptibility and resistance to TZP were defined as MICs of ≤8 and >8 mg/L, respectively, in accordance with the EUCAST breakpoint tables (19). TZP MICs were also measured by broth microdilution method in triplicate at a high inoculum (10^7^ CFU/mL). The inoculum effect was defined as an eightfold MIC increase at higher bacterial density (≥10^7^ vs 10^5^ CFU/mL), as published before (20).

### Spontaneous resistance frequency studies

For each of the initial strains, spontaneous resistance frequency was calculated in triplicate. One colony was incubated in 10 mL of CAMH broth at 37°C for 18 h. After a serial dilution step, 100 µL of each obtained sample was plated on both standard and antibiotic-enriched CAMH (fourfold the initial TZP MIC) agar plates. The spontaneous resistance frequency was defined as the ratio of bacterial count in antibiotic-enriched CAMH agar plate to that of drug-free CAMH agar plate, after 24 h of incubation at 37°C. The lower limit of quantitation (LLOQ) was 1 log_10_ CFU/mL.

### Maximum growth rate (MGR) calculation

For each of the initial strains, maximum growth rate (MGR) was calculated in triplicate. One colony was incubated in 10 mL of CAMH broth at 37°C for 18 h. Then, a standardized solution with an OD measurement of 0.05 at 600 nm was obtained for each strain of interest, and 200 µL was placed in wells of a 96-well plate. Finally, the 96-well plate was incubated for 24 h at 37°C with 108 rpm agitation in the Spark Readr plates with MAGELLAN v3.0 Software (TECAN, Switzerland), with a 600 nm DO measurement every 5 min. All the data were then analyzed by QurvE software (Denmark) to calculate the MGR of the strain of interest, as previously described (21).

### Whole-genome sequencing (WGS)

Short-read WGS was performed by the microbiology platform of the Pasteur Institute (Paris, France), using the Nextera XT DNA library preparation kit (Illumina Inc.) and the NextSeq 500 Sequencing System (Illumina Inc.). After quality assessment using FASTQC, genomes were *de novo* assembled using SKESA (22). Multi-locus sequence typing (MLST) attribution was performed, as well as serotype typing, using appropriate databases (23, 24). Acquired antimicrobial-resistance genes were looked for in the ResFinder database within the FASTQ files using the KMA tool (ResFinder 4.0 for predictions of phenotypes from genotypes). The ratio of gene sequencing was evaluated considering the sequencing depth of two genes of interest with homemade scripts (available on request).

High-molecular-weight genomic DNA was extracted using the Monarch HMW DNA extraction kit (New England Biolabs) and sequenced using Oxford Nanopore Technologies (ONT) MinION with the Native Barcoding Kit 24 V9 (SQK-NBD112.24) on R9.4.1 flow cells (FLO-MIN106D). Basecalling was performed using Guppy v6.5.7 in high-accuracy mode (dna_r9.4.1_450bps_hac.cfg), filtering for mean *Q* score ≥10. Long reads were filtered using Filtlong v0.3.1 (min_length 1,000 bp), and assemblies were generated using Autocycler (v0.6.2) and the Hybracter pipeline (v0.6.0). Plasmid and chromosomal contigs were typed using MOB-suite (v3.1.9), PlasmidFinder (v2.1), and annotated with Prokka (v1.14.6). Acquired resistance genes and copy number variations were determined using ResFinder (v4.1). Base-calling processes, as well as genomic analyses were performed using the *Centre Régional Informatique et d'Applications Numériques de Normandie* (CRIANN) resources and support https://www.criann.fr/.

### Phylogenetic analysis

To assess the genetic relatedness of the three *E. coli* strains, WGS reads were mapped against the *E. coli* ST131 reference genome NCTC13441 (GenBank accession no. HG941718.1) using Snippy (v4.6.0). A core-genome single-nucleotide polymorphism (SNP) alignment was generated, including a contextual regional collection of 392 *E. coli* ST131 clinical genomes collected during genomic surveillance in Normandy, France (https://abrtdb.shinyapps.io/DV_CREM/). Recombination events were detected and removed using Gubbins (v3.3.0). Pairwise SNP distances were computed, and a maximum-likelihood phylogenetic tree was inferred and visualized using iTOL.

### HFIM experiments

The practical aspects of the HFIM have been previously described (17, 25). TZP was infused directly into the central reservoir with an electric syringe (NE-1600, New Era, Darwin Microfluidics, France) controlled by SyringePump PRO software. Cellulosic cartridges (C3008 and C8008; FiberCell Systems, USA) was used (median cutoff of 5 kDa). To ensure concentration equilibrium between all model compartments, the cartridge was connected to the central compartment by a pump (FiberCell Duet Pump, FiberCell Systems, USA) delivering a flow rate >120 mL/min. To simulate piperacillin clearance, two peristaltic pumps (Watson-Marlow 120U/114DV, United Kingdom) continuously renewed CAMH broth within the central compartment.

Each HFIM experiment lasted for 5 days. The extra-capillary space of the cartridge was inoculated at *T* = 0 h with a bacterial load of 10^5^ CFU/mL (low inoculum) or 10^7^ CFU/mL (high inoculum) as it has been previously done in *in vitro* studies assessing the inoculum effect (15, 16). Inoculum was prepared by colony growth in 10 mL of CAMH broth at 37°C overnight.

At 0, 1, 2, 4, 6, 8, 12, 24, 36, 48, 72, 96, and 120 h after inoculation, a 1-mL sample was withdrawn from the extra-capillary space of the cartridge. To minimize carry-over effect, samples were centrifuged twice at 4,000 × *g* for 3 min and resuspended in sterile 0.9% sodium chloride. After a serial-dilution step, 100 µL of the obtained samples was plated on standard and antibiotic-enriched CAMH agar (fourfold the initial TZP MIC) for manual colony counting of total and less-susceptible bacterial populations, respectively, after 24 h of incubation. LLOQ was 1 log_10_ CFU/mL.

Three results were possible at the end of each HFIM experiment. Sustained bacterial suppression was defined as a decrease in total bacterial population ≥3 log_10_ CFU/mL between *T* = 0 h and *T* = 120 h. Bacterial regrowth without resistance emergence was defined as regrowth (difference between bacterial load at 120 and 0 h < 3 log_10_ CFU/mL) observed only on drug-free CAMH agar plates. Finally, bacterial regrowth with resistance emergence was defined as regrowth observed on drug-free and antibiotic-enriched CAMH agar.

### Simulated PK and antibiotic assay

Predicted unbound piperacillin concentration profiles were obtained using Simulix software (Monolix suite, Lixoft, France) and Pmetrics, simulating the PK of a critically ill patient as described by Bergen et al. (total piperacillin clearance = 19 L/h, elimination half-life *t*_1/2_ = 1.4 h) (26). Simulated treatments included two TZP infusion regimens: intermittent (4 g/0.5 g every 6 h over 30 min, free minimal unbound concentration (*fC*_min_) = 6.39 mg/L) and continuous (16 g/2 g over 24 h after a 4 g/0.5 g bolus over 30 min, free concentration at steady state (*fC*ss) = 35.06 mg/mL). The simulated PK and PK/PD targets were assessed for each experimental condition (Table 1).

**TABLE 1:** Simulated pharmacokinetic (PK) and pharmacokinetic/pharmacodynamic (PK/PD) parameters of each HFIM experiment^*a*^

TZP dosing regimen	PK parameters			Baseline TZP MIC (mg/L)	PK/PD parameters				
	*f*AUC_0–24h_ (mg·h/L)	*fC*_max_ (mg/L)	*fC*_min_ or *fC*_ss_ (mg/L)		%*f*T >MIC	%*f*T >4× MIC	%*f*T >8× MIC	fAUC_0–24h_/MIC (h)	*fC*_min/ss_/MIC
Intermittent infusion (4/0.5 g q6h over 30 min)	825	96.98	6.39	2	100%	91%	66.4%	412.50	3.02
				4	100%	66.4%	41.5%	206.25	1.51
				8	91%	41.5%	16.3%	103.13	0.76
Continuous infusion (16/2 g q24h)	961	92.25	35.06	2	100%	100%	100%	480.50	17.53
				4	100%	100%	100%	240.25	8.76
				8	100%	100%	2.1%	120.13	4.38

^*a*^ TZP, piperacillin-tazobactam; fC, free (unbound) concentrations; fCss, free concentration at steady state in continuous infusion.

Samples (1 mL) were withdrawn from the central compartment and stored at −80°C until assayed. The method used for piperacillin and tazobactam determination was high-performance liquid chromatography coupled to tandem mass spectrometry (HPLC-MS/MS). The detailed assay method is described in the supplemental material. The agreement between predicted and observed concentrations for piperacillin and tazobactam, including control conditions without bacteria, was evaluated using the Bland–Altman graphical method, which assesses bias by comparing means and estimating 95% agreement intervals using R (R version 4.3.1, R Foundation for Statistical Computing, Vienna, Austria).

### Characterization of less-susceptible populations

TZP MIC determinations, MGR, and WGS were performed on colonies isolated on antibiotic-containing agar plates (if present) or on drug-free plates (if absent) 120 h after bacterial inoculation, following the methods detailed above. A β-lactamase vs chromosomal ratio was determined according to the sequencing depth.

To assess the phenotypic stability of the less-susceptible populations without antibiotic pressure, one colony of interest was incubated in 10 mL of CAMH broth without antibiotic at 37°C for 24 h, and each day, 200 µL representing ~2 × 10^8^ cells was transferred into fresh CAMH broth for three consecutive days. Each day, ~1.5 × 10^8^ cells were used to perform TZP MIC.

To characterize the genetic context and evaluate potential regulatory or structural mutations in β-lactamase genes, full-length reference sequences encompassing the coding sequences (CDS) and their upstream promoter regions were extracted: a 1,133-bp fragment for bla_CTX-M-15_, including the upstream ISEcp1 promoter region (GenBank accession no. AY044436.1), and a 1,045-bp fragment for bla_TEM-1_, including its native promoter region (GenBank accession no. AL513383.1). Pairwise local alignments against short-read and long-read hybrid assemblies of all parental strains and regrowing isolates were conducted using BLASTn (v2.14.0+; parameters: identity threshold ≥ 90%, query coverage ≥ 60%). Alignments were inspected for single-nucleotide polymorphisms (SNPs), promoter-up variants (including Pa/Pb, P4, and P5 promoters for bla_TEM_), inhibitor-resistant substitutions (IRT mutations), and structural tandem duplications.

## RESULTS

### Strains

Baseline characteristics of each strain: modal TZP MIC at standard and high inoculum, MLST, serotype, spontaneous resistance frequency, MGR, and antibiotic resistance genes are displayed in Table 2. TZP MIC values were highly consistent across replicates, with a maximum variation of one two-fold dilution above or below the modal MIC. Three clinical ESBL-EC strains were studied: *E. coli 1146* (*bla_CTX-M-15_*; TZP MIC = 2 mg/L), *E. coli 3383* (*bla_CTX-M-15_* and *bla_TEM-1B_*; TZP MIC = 4 mg/L), and *E. coli 2262* (*bla_CTX-M-15_* and *bla_OXA-1_*; TZP MIC = 8 mg/L). Long-read assemblies revealed that bla_CTX-M-15_ was integrated into the bacterial chromosome in all three strains, flanked by mobile genetic elements. In strain 3383, bla_TEM-1B_ was located on a conjugative IncFIA/IncFIC plasmid (~292 kb).

**TABLE 2:** Strains phenotypic and genotypic characteristics^*a*^^,^^*b*^

Strain	Multilocus sequence typing	Serotype	Modal TZP MIC (mg/L) at low inoculum (10^5^ CFU/mL)	Modal TZP MIC (mg/L) at high inoculum (10^7^ CFU/mL)	Spontaneous resistance frequency	Maximum growth rate	Antimicrobial resistance genes
*E. coli 1146*	ST 131	O25:H4	2	>256	6.5 × 10^−9^ ± 3.8 × 10^−9^	1.58 ± 0.13	*bla_CTX-M-15_, aadA5, mph(A), sul1, tet(A), dfrA17*
*E. coli 3383*	ST 131	O25:H4	4	>256	1.3 × 10^−8^ ± 9.9 ×10^−9^	1.51 ± 0.10	*bla_CTX-M-15_, bla_TEM-1B_, aph(3″)-Ib, aph(6)-Id, sul2, dfrA14*
*E. coli 2262*	ST 131	O25:H4	8	>256	1.6 × 10^−7^ ± 9.6 × 10^−8^	1.54 ± 0.06	*bla_CTX-M-15_, bla_OXA-1_, aac(3)-IIa, aac(6′)-Ib-cr, aadA5, mph(A), sul1, tet(A*)

^*a*^ Continuous variables are expressed as mean ± standard deviation.

^*b*^ TZP, piperacillin-tazobactam.

### Phylogenetic analysis

High-resolution core-genome SNP analysis revealed substantial genetic divergence among the three strains: *E. coli* 1146 differed from *E. coli* 3383 and *E. coli* 2262 by 39 and 83 SNPs, respectively, while *E. coli* 3383 and *E. coli* 2262 differed by 74 SNPs (Fig. S1). In the phylogenetic reconstruction, each isolate was segregated into distinct clades within the regional ST131 population, demonstrating that they represent independent clinical lineages rather than closely related clonal variants (Fig. S1).

### HFIM pharmacokinetics

Piperacillin and tazobactam free concentration profiles are shown in Fig. S2. Observed and predicted free piperacillin and tazobactam concentrations were significantly correlated (*R*² = 0.88, *P* < 0.001, *R*^2^ = 0.77, *P* < 0.001, respectively). The concordance correlation coefficient (CCC) was 0.93 (95% confidence interval = 0.91–0.95) for piperacillin and 0.83 (95% confidence interval = 0.77–0.88) for tazobactam, indicating substantial agreement between the observed and theoretical measurements. Overall, according to the Bland-Altman figure, the antibiotic concentrations were satisfactory, with the observed values and standard deviations (SDs) closely matching the predicted values (Fig. S3 and S4). Indeed, most values fell within the limits of agreement (±1.96 × SD), except for five measurements for each molecule.

### HFIM experiments

**Fig 1:**
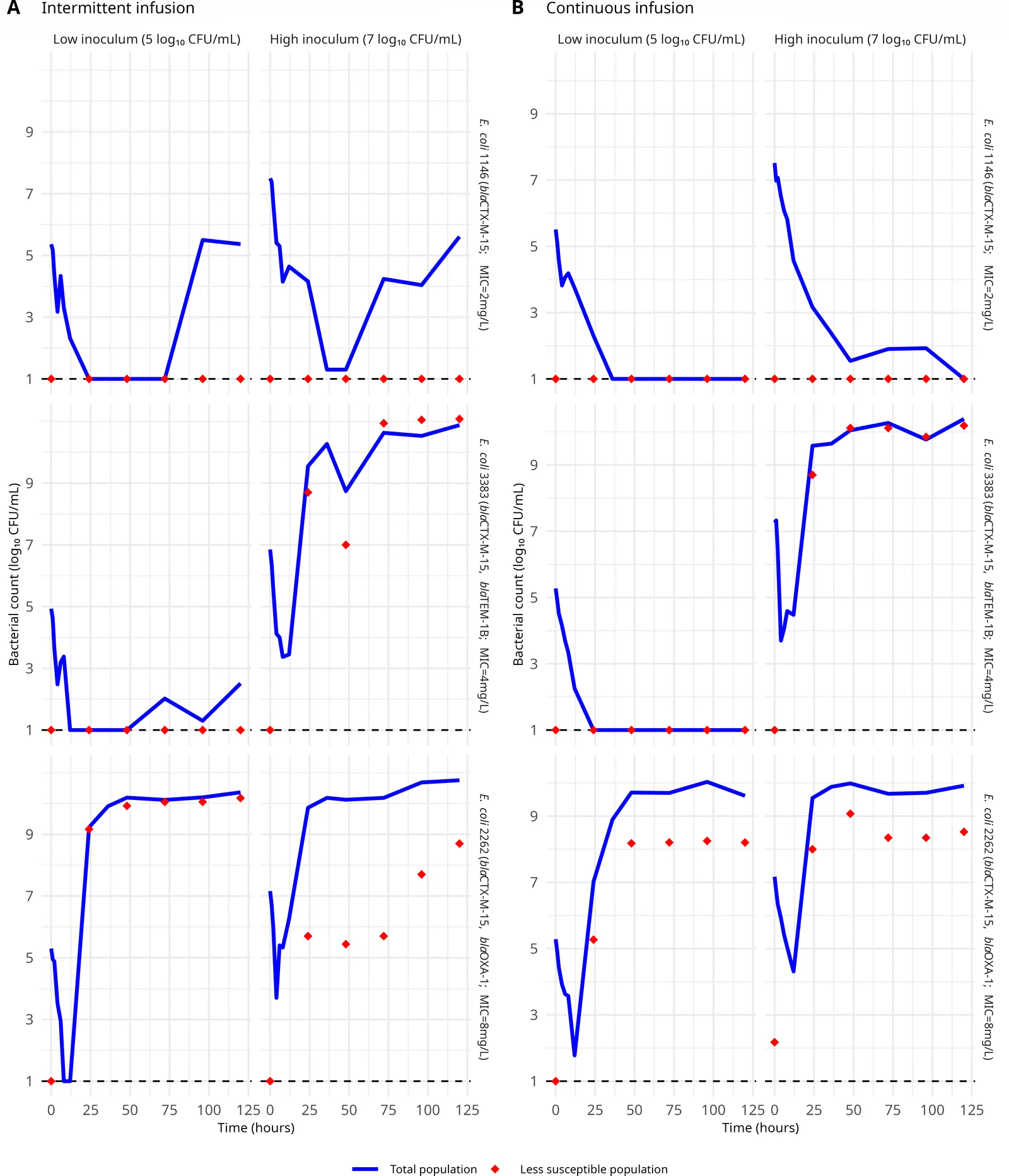
Bacterial count over time in each HFIM experiment according to baseline inoculum, strain, and dosing regimen (**A**: intermittent infution, **B**: continuous infusion). TZP, piperacillin-tazobactam, lower limit of quantitation = 1 log_10_ CFU/mL.

Bacterial killing curves depending on strains and initial inoculum, after 120 h of TZP exposure in intermittent or continuous infusion, are presented in Fig. 1. There was an early decrease in mean viable bacteria for all conditions (−1.92 ± 0.62 log_10_ CFU/mL over 6 h), which does not correspond to bactericidal activity (−3 log_10_ CFU/mL).

Table 3 summarizes the results of all HFIM experiments. For intermittent infusion, regardless of the initial inoculum, strain *2262* (*bla_CTX-M-15_* and *bla_OXA-1_*; TZP MIC = 8 mg/L) exhibited constant and early bacterial regrowth with resistance emergence (TZP MIC > 256 mg/L at 120 h) (+5.06 log_10_ CFU/mL at low inoculum vs +3.59 log_10_ CFU/mL at high inoculum). The same result was observed for continuous infusion (+4.33 log_10_ CFU/mL at low inoculum vs +2.75 log_10_ CFU/mL at high inoculum).

**TABLE 3:** Bacterial count and results of each HFIM experiment according to baseline inoculum^*a*^^,^^*b*^^,^^*c*^

TZP dosing regimen	Strain	Baseline TZP MIC (mg/L)	Bacterial count					
			Low inoculum (10^5^ CFU/mL)			High inoculum (10^7^ CFU/mL)		
			∆ (log_10_ CFU/mL)	Result	Resistance ratio	∆ (log_10_ CFU/mL)	Result	Resistance ratio
Intermittent infusion (4/0.5 g q6h over 30 min)	*E. coli 1146* (*bla_CTX-M-15_*)	2	0	Regrowth	0.186	−1.89	Regrowth	0.178
	*E. coli 3383* (*bla_CTX-M-15_*, *bla_TEM-1B_*)	4	−2.42	Regrowth	0.398	4.04	Regrowth with resistance	1
	*E. coli 2262 *(*bla_CTX-M-15_*, *bla_OXA-1_*)	8	5.06	Regrowth with resistance	0.982	3.59	Regrowth with resistance	0.809
Continuous infusion (16/2 g q24h)	*E. coli 1146* (*bla*_*CTX-M-15*_)	2	−4.51	<LLOQ	0	−6.52	<LLOQ	0
	*E. coli 3383* (*bla_CTX-M-15_*, *bla_TEM-1B_*)	4	−4.28	<LLOQ	0	3.13	Regrowth with resistance	0.981
	*E. coli 2262* (*bla_CTX-M-15_*, *bla_OXA-1_*)	8	4.33	Regrowth with resistance	0.853	2.75	Regrowth with resistance	0.859

^*a*^ ∆ = Bacterial count difference between the end of the HFIM experiment (120 h) and the baseline inoculum.

^*b*^ Resistance ratio defined as the ratio between bacterial count on antibiotic-containing agar plates and that on free-drug agar plates at 120 h.

^*c*^ TZP, piperacillin-tazobactam; LLOQ, lower limit of quantitation (1 log_10_ CFU/mL).

After exposure to intermittent TZP infusion, regrowth with or without resistance emergence was observed at 120 h, regardless of the strain or initial inoculum. Continuous TZP infusion demonstrated sustained bacterial suppression against *3383* strain (*bla_CTX-M-15_* and *bla_TEM-1B_*; TZP MIC = 4 mg/L) at low inoculum and against the *1146* strain (*bla_CTX-M-15_*; TZP MIC = 2 mg/L) at low and high inocula.

The inoculum effect was observed for both infusion regimens. For *3383* strain (*bla_CTX-M-15_* and *bla_TEM-1B_*; TZP MIC = 4 mg/L), intermittent infusion led to regrowth in all inoculum conditions, but with selection and amplification of the resistant population (TZP MIC > 256 mg/L) at high inoculum. Similarly, continuous infusion had contrasting results according to starting inoculum for 3383 strain (*bla_CTX-M-15_* and *bla_TEM-1B_*; TZP MIC = 4 mg/L): sustained bacterial suppression at low inoculum, while regrowth with resistance emergence was observed at high inoculum.

### Characterization of less susceptible population

For all HFIM conditions resulting in regrowth with resistance, TZP MIC for colonies isolated on antibiotic-enriched agar were ≥256 mg/L and phenotypically stable at 72 h (Table 4). As suggested by MGR measurement, no fitness cost was observed in the resistant subpopulation at 120 h compared to parental strain, except for *2262* strain (*bla_CTX-M-15_* and *bla_OXA-1_*; TZP MIC = 8 mg/L) at low starting inoculum exposed to TZP continuous condition (*P* < 0.001).

**TABLE 4:** : Characterization of final subpopulation of each HFIM experiment^*e*^^,^^*f*^

TZP dosing regimen	Strain	Baseline TZP MIC (mg/L)	Final subpopulation modal TZP MIC (mg/L)^*a*^		*bla*_CTX-M-15_ vs chromosome ratio^*b*^		Maximum growth rate (MGR)		MGR comparison to parental strain, *P* value	
			Low inoculum (10^5^ CFU/mL)	High inoculum (10^7^ CFU/mL)	Low inoculum (10^5^ CFU/mL)	High inoculum (10^7^ CFU/mL)	Low inoculum (10^5^ CFU/mL)	High inoculum (10^7^ CFU/mL)	Low inoculum (10^5^ CFU/mL)	High inoculum (10^7^ CFU/mL)
Intermittent infusion (4/0.5 g q6h over 30 min)	*E. coli 1146* (*bla_CTX-M-15_*)	2	2^*c*^	4^*c*^	2	2	NA	NA	NA	NA
	*E. coli 3383* (*bla_CTX-M-15_*, *bla_TEM-1B_*)	4	4^*c*^	>256	2.5^*d*^	89^*d*^	NA	0.85 ± 0.11	NA	<0.001
	*E. coli 2262* (*bla_CTX-M-15_*, *bla_OXA-1_*)	8	>256	256	28.9	6.5	1.57 ± 0.09	1.33 ± 0.08	0.150	<0.001
Continuous infusion (16/2 g q24h)	*E. coli 1146* (*bla_CTX-M-15_*)	2	<LLOQ^*g*^	<LLOQ^*g*^	NA	NA	NA	NA	NA	NA
	*E. coli 3383* (*bla_CTX-M-15_*, *bla_TEM-1B_*)	4	<LLOQ^*g*^	>256	NA	53^*d*^	NA	1.54 ± 0.10	NA	0.212
	*E. coli 2262* (*bla_CTX-M-15_*, *bla_OXA-1_*)	8	>256	256	18	11	1.62 ± 0.06	1.57 ± 0.16	<0.001	0.376

^*a*^ Final subpopulation TZP MICs were determined on isolates growing on antibiotic-enriched agar plates 120 h after inoculation.

^*b*^ β-lactamase vs chromosomal ratio was determined according to the sequencing depth.

^*c*^ TZP MIC determination on isolates growing on standard agar plates.

^*d*^ Due to chromosomal integration of the bla_CTX-M-15_ gene, the elevated β-lactamase was the bla_TEM-1_ gene for this strain.

^*e*^ MGR are expressed as mean ± standard deviation and were compared by *t*-test.

^*f*^ TZP, piperacillin-tazobactam; LLOQ, lower limit of quantitation (1 log_10_ CFU/mL); NA, not available.

^*g*^ <LLOQ meaning that no isolates grow on standard or antibiotic-enriched agar 120 h after inoculation (total bacterial suppression).

Genomic analysis of the resistant subpopulation at 120 h compared to parental strain demonstrated targeted gene amplification events driving high-level TZP resistance. In strain 2262 (bla_CTX-M-15_ and bla_OXA-1_), multiple tandem duplications of the bla_CTX-M-15_ locus (five to seven repeat copies, 3.93 kb repeat units) were consistently identified across all regrowing isolates. In strain 3383 regrowing under continuous TZP infusion at high inoculum, a massive tandem duplication of the plasmid-borne bla_TEM-1B_ region occurred, yielding 13 copies of the bla_TEM-1B_ cassette (3.97 kb repeat units). In strain 3383 regrowing under intermittent TZP infusion at high inoculum, short-read read-depth analysis revealed an 88-fold increase in bla_TEM-1B_ coverage relative to the chromosomal mean, consistent with substantial gene amplification. Although the long-read hybrid assembly reconstructed a single consensus unit on a fragmented plasmid contig (42.8 kb), a known artifact of *de novo* assemblers collapsing large, identical tandem repeats, the read-depth quantification combined with the fully resolved 13-copy tandem array confirms that high-level TEM-1B hyperproduction via gene amplification is the shared mechanism of regrowth across high-inoculum regimens for strain 3383.

For bla_CTX-M-15_, all parental strains and regrowing isolates exhibited 100.00% sequence identity (0 substitutions, 0 gaps) across the entire promoter and coding region. Similarly, all repeat units within the tandem amplification arrays in strain 2262 and its derivatives were identical.

For bla_TEM-1B_, sequence identity between the parental strain 3383 and the regrowing isolates was 100.00% with respect to the wild-type bla_TEM-1B_ allele (harboring only the synonymous T395G marker relative to blaTEM-1A). Crucially, all 13 repeated copies in the tandem array of strain 3383 regrowing under continuous TZP infusion at high inoculum maintained 100.00% identity without any promoter mutations (such as Pa/Pb or P4/P5 promoter-up variants) or inhibitor-resistant TEM (IRT) substitutions. These results demonstrate that the observed resistance phenotypes are exclusively mediated by gene dosage amplification rather than transcriptional promoter upregulation or structural enzyme modifications. All the results are available in Table S1.

## DISCUSSION

In this study, we explored how PK/PD optimization may overcome the inoculum effect affecting TZP *in vitro* activity against severe infection due to three ESBL-EC with TZP MICs of 2, 4, and 8 mg/L. Our HFIM results showed that TZP intermittent infusion is likely ineffective against ESBL-EC infections independent of initial inoculum or TZP MIC. PK/PD optimization through continuous infusion overcame the inoculum effect against the strain with a TZP MIC ≤ 2 mg/L (*bla_CTX-M-15_*) regardless of inoculum size, and against the strain with a TZP MIC of 4 mg/L (*bla_CTX-M-15_* and *bla_TEM-1B_*) at low initial inoculum only. Continuous infusion consistently failed at high inoculum for the strain with a TZP MIC of 4 mg/L (*bla_CTX-M-15_* and *bla_TEM-1B_*), and at any inoculum for TZP MIC of 8 mg/L (*bla_CTX-M-15_* and *bla_OXA-1_*).

Despite achieving ~100% fT>MIC, intermittent infusion failed to reach the 100%fT >4× MIC threshold recommended for critically ill patients (11), which is consistent with the systematic observed regrowth for all strains. Previous HFIM studies on ESBL-E consistently observed bacterial regrowth with resistance emergence after exposure to TZP intermittent infusion (27). Consistent with these experimental data, a meta-analysis of clinical studies in intensive care unit (ICU) patients with documented or suspected gram-negative infections, whatever the inoculum size, found that reaching a 100%fT >4–5× MIC threshold was independently associated with clinical cure (OR = 1.69; 95% confidence interval = 1.15–2.49), further supporting the inadequacy of standard intermittent dosing in this population (28). These findings emphasize the need for PK/PD optimization through continuous infusion, whose efficacy against ESBL-E was, however, conditional on both MIC and inoculum size.

Loading dose followed by continuous infusion systematically achieved sustained bacterial suppression against strain *1146* (*bla_CTX-M-15_*; TZP MIC = 2 mg/L) at both low and high inocula, suggesting that PK/PD optimization fully overcame the inoculum effect at this MIC level. This could be explained by the absence of β-lactamase co-production (OXA-1) and the spontaneous resistance frequency of 10^−9^, resulting in a probability of a pre-existing less-susceptible population below 1 cell even at high inoculum (10^7^ CFU/mL) and preventing resistance amplification regardless of the bacterial load.

This favorable outcome was not sustained for strain *3383* (*bla_CTX-M-15_* and *bla_TEM-1B_*; TZP MIC = 4 mg/L) at high inoculum, where continuous infusion failed to prevent resistance emergence. This discrepancy may be explained by the intrinsic characteristic of this strain, such as its mutation frequency (1.3 × 10^−8^). Indeed, it was close to the initial high inoculum (10^7^ CFU/mL), thus increasing the probability of a pre-existing less-susceptible population at high inoculum, which could be selected and amplified early under antibiotic treatment. A similar mechanism was found in another study using HFIM and mathematical modeling to describe TZP efficacy against *Pseudomonas aeruginosa* infection (29). Felton et al. demonstrated that with a mutation frequency of 10^−7^ and an initial inoculum ≥10^7^ CFU/mL, at least one pre-existing cell is statistically inevitable, leading to resistance amplification regardless of the infusion mode.

For the strain *2262* (*bla_CTX-M-15_* and *bla_OXA-1_;* TZP MIC = 8 mg/L), treatment failure was observed regardless of the inoculum size, suggesting that mechanisms beyond inoculum density were responsible. Treatment failure occurred despite optimal PK/PD target attainment (100%fT >4× MIC with continuous infusion) and a low mutation frequency, pointing to an intrinsic mechanism of resistance rather than a PK/PD or inoculum-related failure. Besides CTX-M-15, this strain produced OXA-1 β-lactamase, which is not inhibited by tazobactam (30). Furthermore, OXA-1 co-production was statistically associated with higher mortality rates in patients treated with TZP, compared to carbapenems in a *post hoc* analysis of the MERINO study (10). OXA-1 co-production may be suspected in cases of phenotypic resistance to amoxicillin/clavulanate (31) associated with elevated TZP MIC (between 8 and 32 mg/L) (32). It should be noted that this OXA-1/ESBL co-production is common (up to 42% of ESBL-E) (32), but no pathognomonic phenotypic markers currently exist, making its detection difficult. Taken together, these findings suggest that the clinical relevance of TZP in ESBL-EC infections depends on a combination of MIC, inoculum size, and β-lactamase profile. Nevertheless, as strain 2262, despite the highest MIC, the direct implication of OXA-1 production on failure would require specific studies.

A striking finding from our genomic analysis was the identification of rapid tandem gene duplications driving high-level TZP resistance in regrowing isolates. Rather than acquiring novel coding or promoter mutations, clinical isolates subjected to high bacterial densities (7 log_10_ CFU/mL) adapted via gene dosage amplification. Indeed, we observed a massive amplification of the plasmid-borne bla_TEM-1B_ cassette (up to 13 tandem repeat units) in strain 3383 (*bla_CTX-M-15_* and *bla_TEM-1B_*; TZP MIC = 4 mg/L) regrowing under continuous infusion at high inoculum and multiple tandem duplications of bla_CTX-M-15_ in isolate derivatives from strain 2262 (*bla_CTX-M-15_* and *bla_OXA-1_*; TZP MIC = 8 mg/L). Tandem gene duplication and amplification are established drivers of phenotypic heteroresistance under β-lactam/β-lactamase inhibitor exposure (33, 34). Consistent with our findings, Dubey et al. recently showed, in an HFS, that exposure of *E. coli* to another β-lactam/β-lactamase inhibitor combination (amoxicillin-clavulanate) selected pre-existing resistant subpopulations through IS-mediated *bla_TEM-1_* amplification and β-lactamase hyperproduction (35). This supports gene-dosage amplification as a rapidly selectable resistance mechanism under β-lactam/β-lactamase inhibitor pressure. Moreover, mobile genetic elements, such as insertion sequences, can mediate unequal homologous recombination to generate head-to-tail arrays that rapidly expand gene dosage (36). At low initial inoculum, the fixed tazobactam concentration is sufficient to inhibit baseline enzyme levels. In contrast, at high initial bacterial loads, the pre-existing subpopulation carrying spontaneous duplications is statistically larger. Under TZP selective pressure, these amplified arrays expand, resulting in β-lactamase hyperproduction that stoichiometrically overwhelms and titrates out the available tazobactam (20, 37). This mechanism provides a clear molecular basis for the pronounced inoculum effect observed in our HFIM experiments, where continuous infusion succeeded at low inoculum but failed at high inoculum for strain 3383 (*bla_CTX-M-15_* and *bla_TEM-1B_*; TZP MIC = 4 mg/L). Crucially, standard binary genotypic prediction pipelines (relying solely on gene presence or absence) overlook these copy-number dynamics and fail to anticipate rapid in-host failure unless quantitative read-depth metrics or structural long-read sequencing are applied (36, 37). In severe, high-bacterial-load infections, the propensity for rapid *in vivo* β-lactamase amplification constitutes a major limitation to using TZP as a carbapenem-sparing regimen, even when initial MICs fall within the susceptible range (10).

When TZP is considered as a carbapenem-sparing agent in ICU patients, continuous infusion may be strongly preferred, as supported by the BLING III randomized trial demonstrating improved clinical cure rate (absolute difference: 5.7%; 95% confidence interval = 2.4–9.1) with continuous β-lactam infusion in 7,031 ICU patients (38). However, its continuous use should be further restricted based on MIC and estimated inoculum. When the clinical source of infection is known, and a low inoculum is suspected, such as in urinary tract infection (UTI) or biliary infection, continuous infusion of TZP could be an alternative option for ESBL-EC if MIC ≤ 4 mg/L. In case of high bacterial density, typically for ventilator-associated pneumonia, continuous infusion of TZP should be used only for ESBL-EC strains with a TZP MIC ≤ 2 mg/L. This inoculum-based stratification is supported by a meta-analysis showing that mortality was significantly higher with TZP vs carbapenems specifically in cohorts including fewer than 50% of UTI/biliary infection (OR = 2.02; 95% confidence interval = 1.00–4.07) (28). The MIC threshold proposed here (≤4 mg/L at low inoculum, ≤2 mg/L at high inoculum) is derived from our experimental data and should not be interpreted as definitive guidance. Further clinical work should explore our hypothesis.

This study has several limitations. HFIM, as an *in vitro* model, lacks host immune response and may underestimate clinical efficacy of TZP. However, due to this feature, HFIM only reflects interaction between drug and bacteria, which may be particularly relevant for ICU patients with sepsis-induced immune dysfunction (39), though immune response remains variable in this population. Furthermore, the HFIM experiments were not replicates. We studied only three *E. coli* clinical strains that harbor the most prevalent ESBL gene carried by Enterobacterales but in a specific genetic background (a chromosomally integrated *bla_CTX-M-15_*); therefore, our results could not be generalized to other Enterobacterales (40). Future work should evaluate whether similar inoculum-dependent treatment failures and β-lactamase amplification mechanisms are observed in strains carrying plasmid-borne ESBL genes. Additionally, the two inoculum sizes tested may not fully capture the spectrum of bacterial densities encountered in clinical infections, particularly in high-inoculum sources such as abscesses.

In conclusion, this study provides experimental evidence that PK/PD optimization through continuous infusion can overcome the inoculum effect in ESBL-EC infection under specific conditions. Continuous infusion should therefore be preferred when TZP is used in ICU patients. However, the strategy was effective only for isolates with a TZP MIC < 4 mg/L, which was not overcome by PK/PD optimization. Our experimental results suggest a mechanism-guided, personalized approach when considering TZP as a carbapenem-sparing agent in critically ill patients, integrating both susceptibility results, expected inoculum, and β-lactamase profile. Further *in vivo* and clinical research are needed to consolidate these findings.

## Data Availability

Sequencing data generated in this study have been deposited in the European Nucleotide Archive (ENA) under the following accession code: PROJECT PRJEB126846.

## SUPPLEMENTAL MATERIAL

Supplemental figures and tables spectrum.01457-26-s0001.docx Fig. S1 to S4 and Table S1.

ASM does not own the copyrights to Supplemental Material that may be linked to, or accessed through, an article. The authors have granted ASM a non-exclusive, world-wide license to publish the Supplemental Material files. Please contact the corresponding author directly for reuse.
