# Supplementary material for: Impact of inoculum effect on in vitro activity of intermittent and continuous piperacillin-tazobactam infusion against ESBL-producing Escherichia coli in a hollow-fiber infection model
Source: Microbiol Spectr. 2026 Oct 13;14(11):e01457-26. doi: 10.1128/spectrum.01457-26 (PMC13729154; doi:10.1128/spectrum.01457-26)
Supplement: Supplementary file 1 [file spectrum.01457-26-s0001.docx]

**SUPPLEMENTARY MATERIAL**

*Phylogenetic analysis*

**Figure S1:** Phylogenetic reconstruction of the three isolates (*E. coli* 1146, 3383 and 2262) compared to *E. coli* ST131 reference genome NCTC13441 and to regional collection of 392 *E. coli* ST131 clinical genomes.

*Piperacillin and tazobactam assay*

One-millilitre samples were withdrawn from the central reservoir at 0.5, 6, 6.5, 12, 12.5, 24, 24.5, 48, 48.5, 72, 72.5, 96, 96.5 and 120 h after inoculation for the intermittent infusion regimens, and at time 0.5, 2, 4, 6, 8, 24, 48, 72, 96 and 120 h for the continuous infusion regimens.

Samples were analysed after protein precipitation in methanol, in the presence of deuterated isotopes of each analyte, used as internal standards. Chromatographic separation was carried out in gradient mode on a C^18^-type column, and quantification in Multiple Reaction Monitoring (MRM) mode on a 4500QTRAP mass spectrometer (Shimadzu, Kyoto, Japan). The ion transitions used were m/z 517.907 → 143 for the quantification step and m/z 517.907 → 115 for the confirmation step.

**Figure S2:** Observed (points) and predicted (lines) free piperacillin and tazobactam concentrations over time, for intermittent (A) and continuous (B) infusion regimens.
*All data displayed correspond to a bacterial inoculum lower or equal to 7 log_10_ CFU/mL at the time of sampling.*

**Figure S3:** Agreement between predicted and observed piperacillin concentrations in Bland–Altman graphical method.
*All data displayed correspond to a bacterial inoculum lower or equal to 7 log_10_ CFU/mL at the time of sampling.*

*Mean difference (black dotted line) and limits of agreement defined as mean ± 1.96 × standard deviation (red lines) with 95% confidence intervals (purple shaded areas).*

**Figure S4:** Agreement between predicted and observed tazobactam concentrations in Bland–Altman graphical method.
*All data displayed correspond to a bacterial inoculum lower or equal to 7 log_10_ CFU/mL at the time of sampling.*

*Mean difference (black dotted line) and limits of agreement defined as mean ± 1.96 × standard deviation (red lines) with 95% confidence intervals (purple shaded areas).*

**Table S1:** BLAST alignment results of β-lactamase genes (blaCTX-M-15, blaTEM-1B) in parental strains and resistant subpopulations recovered at 120 h, including gene localization, copy number, sequence identity, and substitution details.
